# Temporal and spatial trends in gastric cancer burden in the USA from 1990 to 2021: findings from the global burden of disease study 2021

**DOI:** 10.3389/fonc.2024.1499384

**Published:** 2024-12-18

**Authors:** Chengwei Zhan, Binxu Qiu, Jun Wang, Yanhua Li, Jinhai Yu

**Affiliations:** ^1^ Daytime Observation Unit, The First Hospital of Jilin University, Changchun, Jilin, China; ^2^ Department of Laboratory Medicine, Med+X Center for Manufacturing, West China Hospital, Sichuan University, Chengdu, Sichuan, China; ^3^ Department of General Surgery, National Clinical Research Center for Geriatrics, West China Hospital, Sichuan University, Chengdu, Sichuan, China; ^4^ Breast Center, West China Hospital, Sichuan University, Chengdu, Sichuan, China; ^5^ Department of Critical Care Medicine, The First Hospital of Harbin Medical University, Harbin, China; ^6^ Department of Gastric and Colorectal Surgery, General Surgery Center, The First Hospital of Jilin University, Changchun, Jilin, China

**Keywords:** GC, age-period-cohort analysis, ARIMA model, disease burden, join-point regression

## Abstract

**Background:**

Gastric cancer (GC) is a significant public health concern in the USA, and its burden is on the rise.

**Methods:**

This study utilized the latest data from the Global Burden of Disease (GBD) study. We provided descriptive statistics on the incidence, prevalence, mortality, disability-adjusted life years (DALYs), and age-standardized rates (ASRs) of GC across the USA and states. By calculating percentage changes and average annual percentage changes (AAPC), along with conducting age-period-cohort analysis, we assessed the trends in the burden of GC. Decomposition analysis was then performed, followed by the application of an autoregressive integrated moving average (ARIMA) model to forecast changes in ASRs through 2036.

**Results:**

From 1990 to 2021, the number of incidence and prevalence of GC in the USA increased, but age-standardized incidence rates (ASIR) trended downward (AAPC = -0.73, 95% confidence interval [CI]: -0.77 to -0.68) and age-standardized prevalence rates (ASPR) (AAPC = -0.99, 95% CI: -1.08 to -0.9) showed a decreasing trend. In addition, the number of deaths, DALYs, age-standardized mortality rates (ASMR) and age-standardized DALYs rates (ASDR) in GC showed a decreasing trend. The burden of GC was significantly higher in males compared to females. In addition, we found that the highest incidence and prevalence in females was in the age group of 75-79 years, whereas the highest incidence and prevalence in males was in the age group of 70-74 years.

**Conclusion:**

GC is a major public health issue in the USA. Although ASIR, ASPR, ASMR, and ASDR for GC are decreasing, the number of incidence and prevalence of GC in the USA remains high, and the disease burden of GC in the USA remains high. Strengthening preventive interventions, particularly for men and patients over the age of 60, will be crucial in the future.

## Introduction

The American Cancer Society estimates that in 2023, there will be approximately 26,500 new cases of gastric cancer (GC) in the USA, with about 11,130 deaths attributed to this disease (1). Although the incidence of GC has steadily declined over the past few decades, and the mortality rate decreased by 41.5% between 1990 and 2017, GC remains a significant public health concern (2). This improvement is largely due to advancements in diagnostic techniques, better control of Helicobacter pylori (Hp) infections, and changes in dietary habits (3). However, GC continues to pose a substantial burden, particularly among certain population groups and geographic regions. In the USA, if GC is detected at an early stage, the 5-year survival rate is approximately 70%. However, about 60% of cases are diagnosed at a regional or distant stage, at which point the 5-year survival rates drop significantly to 31% and 6%, respectively (4, 5).

GC exhibits significant epidemiological heterogeneity, with incidence rates differing by 5 to 10-fold between high-risk and low-risk regions (2). This pronounced geographic variation can be partly attributed to differences in Hp infection rates across populations, a well-established and significant risk factor for GC (6). Additionally, once infected with Hp, certain lifestyle and dietary habits can substantially increase the risk of developing GC. For instance, high alcohol consumption, the intake of processed meats, and a diet deficient in fresh fruits and vegetables are all closely associated with an elevated risk of GC (7, 8). In the USA, low-income populations, particularly those in economically underdeveloped central regions, continue to face challenges in accessing healthy food options, making them more vulnerable to poor dietary quality (9, 10). Besides Hp infection, other environmental factors, such as smoking and socioeconomic status, also play critical roles in influencing the risk of GC (11, 12).

The objective of the study is to comprehensively analyze the temporal trends of GC in the USA using data from the Global Burden of Disease (GBD) 2021 study. By examining incidence, prevalence, mortality, and disability-adjusted life years (DALYs) across different states, age groups, and genders, the study aims to provide a detailed understanding of the burden of GC in the USA and identify key factors contributing to regional disparities. Ultimately, the research seeks to inform public health strategies that can mitigate the impact of GC through targeted prevention and intervention efforts.

## Materials and methods

### Data source

The burden of GC in the USA from 1990 to 2021 is sourced from the Global Health Data Exchange 2021 results website (https://vizhub.healthdata.org/gbd-results/) (13). GBD 2021 offers estimates for incidence, prevalence, mortality, and DALYs related to 371 diseases and injuries across 204 countries and regions, along with data on gender and 87 risk factors (14). It comprises a total of 86,249 data sources from censuses, household surveys, civil registration and vital statistics, disease registries, health service utilization, air pollution monitoring, satellite imaging, disease notification and other sources. Each dataset is individually standardized, with a key step involving the coding of diseases according to the International Classification of Diseases (ICD). The reliability and representativeness of the data have been validated through various studies using the USA GBD data. Mortality rates due to GC from 1990 to 2021 were simulated using vital registration, verbal autopsy data, and surveillance system data. The data were standardized and mapped according to the GBD cause of death ICD mapping methodology, which assigns each death to a single underlying cause of death. Ethical approval is not required as human subjects are not directly involved. This study adheres to the STROBE criteria (15).

### Join-point regression model

The Joinpoint regression analysis was employed to examine trends in age-standardized incidence rates (ASIR), age-standardized prevalence rates (ASPR), age-standardized mortality rates (ASMR), and age-standardized DALYs rates (ASDR) from 1990 to 2021. The analysis began with the collection and preparation of time series data, such as annual ASIR, ASPR, ASMR, and ASDR. Exploratory analyses were performed to identify overall trends and potential inflection points. The Joinpoint regression model accommodates multiple potential inflection points, segmenting the time series into distinct linear segments. Each segment’s slope represents the annual percent change (APC) for that period (16). Following the selection of the optimal model, the trend and location of each inflection point were described, and the APC for each segment was calculated. Additionally, the average annual percent change (AAPC) was computed to provide a comprehensive view of long-term trends by integrating multiple APCs.

### Age-Period-Cohort model

The Age-Period-Cohort model is a statistical framework used to analyze the effects of age, time period, and birth cohort on disease incidence and mortality rates. This model decomposes these three factors to understand their separate impacts on health outcomes. Age effects refer to variations in disease risk as individuals age, while period effects capture temporal changes that affect all age groups simultaneously. Cohort effects reveal differences in disease patterns among different birth cohorts, reflecting generational influences (17, 18). In our study, the model was employed to dissect trends in GC incidence, prevalence, mortality, and DALYs in the USA. This approach allows for a comprehensive understanding of how aging populations, temporal shifts, and generational factors contribute to changes in disease burden over time.

### Decomposition analysis

The Das Gupta method of decomposition analysis is an epidemiological technique used to identify the factors driving changes in disease burden over time (19, 20). This method breaks down changes in incident cases, prevalence cases, mortality cases, and DALYs into three primary components: epidemiological changes, population growth, and population aging. Epidemiological changes refer to variations in disease incidence or mortality rates, reflecting improvements in medical technology and public health. Population growth pertains to changes in the overall population that affect disease burden, where rapid population increases can amplify disease burden even if incidence and mortality rates remain constant. Population aging refers to the phenomenon where an increasing proportion of elderly individuals within the population may lead to a higher burden of chronic and non-communicable diseases.

### Autoregressive integrated moving average model

The ARIMA model is a widely employed tool in time series analysis, particularly useful for GBD research (21, 22). It incorporates three components: Autoregressive, Integrated, and Moving Average, to model and forecast time-dependent data. The parameters of the ARIMA model include the number of autoregressive terms, the number of differences required to achieve stationarity, and the number of moving average terms. The model assumes that the data sequence is stationary, necessitating the transformation of non-stationary data into a stationary format. Parameter estimation is performed using the Autocorrelation Function and Partial Autocorrelation Function, and model selection is guided by the Akaike Information Criterion and the Bayesian Information Criterion.

### Statistical analysis

The Joinpoint regression analyses were performed by using Joinpoint software (version 5.2.0.0) and other analyses were performed using R (version 4.3.1). Statistical significance was defined as a two-sided P value of less than 0.05.

## Result

### Comparison of GC burden between the USA and the global

In 2021, the number of GC-related incident cases, prevalent cases, deaths, and DALYs in the USA were estimated at 28,458.22 (95% uncertainty interval (UI): 26,390.25–29,870.53), 74,986.82 (95% UI: 70,463.87–78,357.48), 16,444.97 (95% UI: 15,029.59–17,351.08), and 363,139.06 (95% UI: 342,100.1–377,924.87), respectively. These figures represent 2.31%, 3.13%, 1.72%, and 1.59% of the global totals, respectively (**Supplementary Table S1**). The ASIR, ASPR, ASMR, and ASDR for GC in the USA were 5.07 per 100,000 population (95% UI: 4.74–5.3), 13.88 per 100,000 population (95% UI: 13.12–14.47), 2.84 per 100,000 population (95% UI: 2.62–2.98), and 69.16 per 100,000 population (95% UI: 65.84–71.77), respectively. Notably, these rates were significantly lower than the global averages, representing 0.35, 0.5, 0.25, and 0.26 times the global ASIR, ASPR, ASMR, and ASDR, respectively (**Supplementary Table S2**).

From 1990 to 2021, the global number of new GC cases, prevalent cases, deaths, and DALYs increased by 25.42%, 43.2%, 11.73%, and -1.94%, respectively, while the corresponding figures for the USA showed increases of 10.69%, 33.59%, -5.03%, and -5.22%, respectively, all of which were lower than the global levels (**Supplementary Table S3**). From 1990 to 2021, the AAPCs in the global ASIR, ASPR, ASMR, and ASDR for GC were -1.77 (95% confidence interval [CI]: -1.91 to -1.63), -1.26 (95% CI: -1.4 to -1.12), -2.17 (95% CI: -2.28 to -2.06), and -2.42 (95% CI: -2.52 to -2.33), respectively. In comparison, the corresponding AAPCs in the USA were -1.51 (95% CI: -1.64 to -1.37), -0.83 (95% CI: -1.14 to -0.53), -2.07 (95% CI: -2.19 to -1.96), and -1.91 (95% CI: -1.99 to -1.83) (**Supplementary Table S4**).

### Burden of GC across USA states

**Figure 1:**
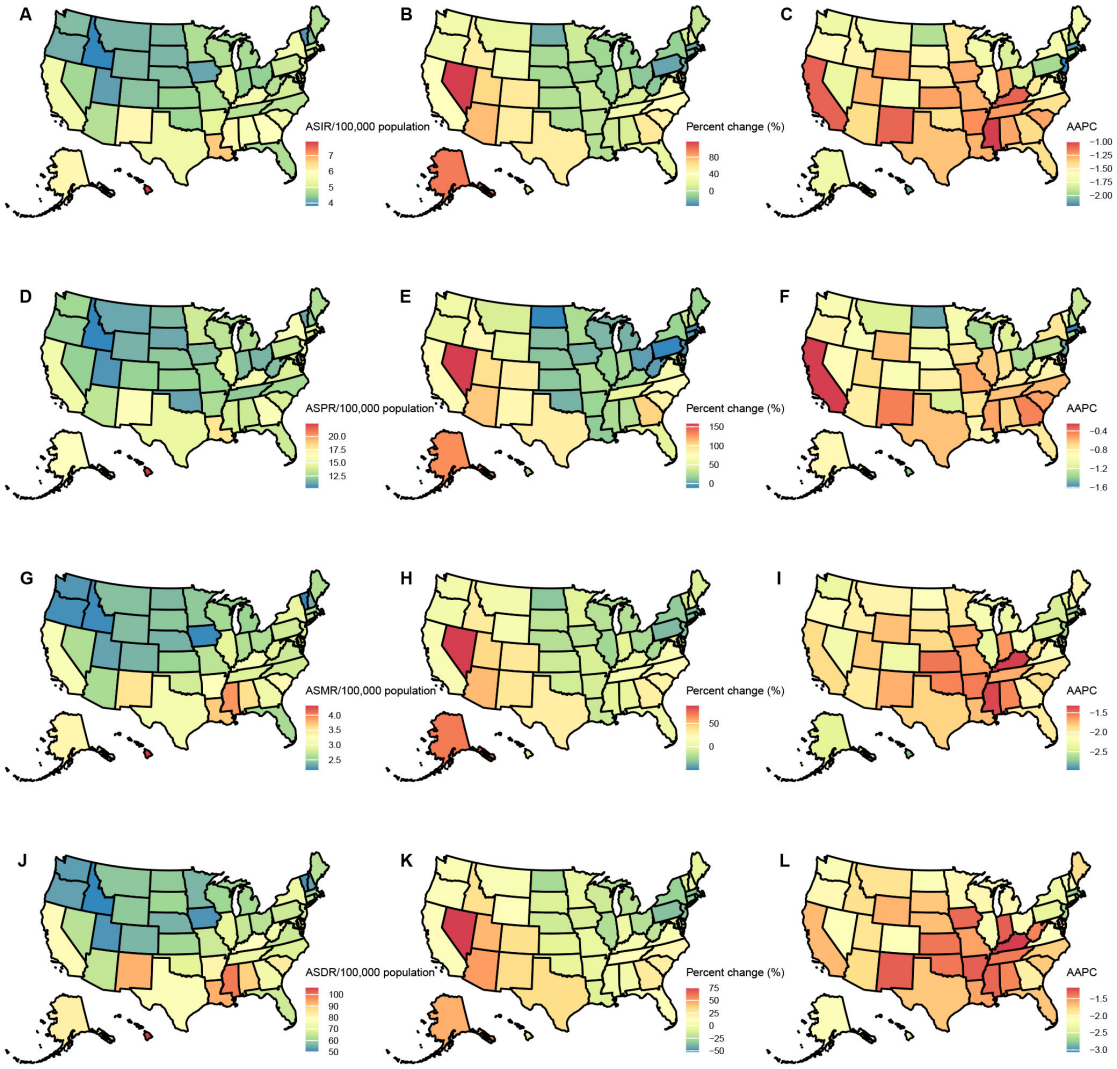
Age-standardized rates of GC burden in 2021 and the temporal trends from 1990 to 2021 by states in the USA. **(A)** ASIR in 2021; **(B)** Percent change in ASIR from 1990 to 2021; **(C)** AAPC in ASIR from 1990 to 2021; **(D)** ASPR in 2021; **(E)** Percent change in ASPR from 1990 to 2021; **(F)** AAPC in ASPR from 1990 to 2021; **(G)** ASMR in 2021; **(H)** Percent change in ASMR from 1990 to 2021; **(I)** AAPC in ASMR from 1990 to 2021; **(J)** ASDR in 2021; **(K)** Percent change in ASDR from 1990 to 2021; **(L)** AAPC in ASDR from 1990 to 2021. ASIR: age-standardized incidence rate; ASPR: age-standardized prevalence rate; ASMR, age-standardized mortality rate; ASDR, age-standardized DALYs rate; AAPC, estimated annual percentage change; GC, thyroid cancer.

Among USA states, the highest numbers of GC incident cases, prevalent cases, deaths, and DALYs were observed in California, Texas, Florida, New York, and Pennsylvania. In contrast, the states with the lowest numbers were the District of Columbia, Wyoming, Vermont, North Dakota, and Alaska. The states with the highest ASIR and ASPR were Louisiana, Hawaii, New Mexico, and Alaska, with Mississippi also ranking high in ASIR (6.05, 95% UI: 5.09–7.22) and New Jersey in ASPR (16.63, 95% UI: 13.62–19.85). Idaho, Utah, and Vermont consistently ranked among the states with the lowest ASIR and ASPR, with Iowa (ASIR: 4.1, 95% UI: 3.37–4.86) and Oregon (ASIR: 4.17, 95% UI: 3.47–5.01) joining the lowest ASIR group, while Oklahoma (ASPR: 11, 95% UI: 9.29–13.15) and Montana (ASPR: 11.06, 95% UI: 9.38–13.25) were included in the lowest ASPR group. For ASMR and ASDR, the states with the highest rates were Hawaii, Mississippi, Louisiana, and the District of Columbia, with Alabama (ASMR: 3.57, 95% UI: 3.08–4.14) and New Mexico (ASDR: 92.65, 95% UI: 77.82–108.62) also ranking high. Conversely, the states with the lowest ASMR and ASDR were Vermont, Iowa, Idaho, and Washington, with Oregon (ASMR: 2.2, 95% UI: 1.83–2.63) and Utah (ASDR: 51.56, 95% UI: 43.53–59.74) also among the lowest. This study provides a detailed analysis of the GC burden across USA states. From 1990 to 2021, the states with the greatest increases in incident and prevalent cases were Nevada, Arizona, Utah, and Alaska, while the smallest increases were observed in the District of Columbia. The largest increases in deaths and DALYs occurred in Nevada, Arizona, Utah, Alaska, and New Mexico, with the District of Columbia again showing the smallest increases. The District of Columbia also experienced the most significant reductions in ASIR, ASPR, ASMR, and ASDR (**Tables 1**–**4**, **Figure 1**).

**Table 1:** Incident cases and ASIR of gastric cancer between 1990 and 2021 by gender and states.

Characteristics	Incident cases			ASIR per 100,000		
	1990 N (95% UI)	2021 N (95% UI)	Percentage change (%)	1990 N (95% UI)	2021 N (95% UI)	AAPC (95% CI))
**United States of America**	25710.59 (24105.79-26591.06)	28458.22 (26390.25-29870.53)	10.69%	8.04 (7.57-8.3)	5.07 (4.74-5.3)	-1.51 (-1.64 to -1.37)
Sex						
Female	10129.4 (9165.06-10665.15)	11003.74 (9802.04-11707.83)	8.63%	5.31 (4.88-5.55)	3.6 (3.27-3.8)	-1.24 (-1.37 to -1.11)
Male	15581.18 (14920.82-16031.01)	17454.48 (16328.03-18202.32)	12.02%	11.7 (11.17-12.05)	6.79 (6.37-7.07)	-1.79 (-1.92 to -1.67)
State						
Alabama	436.77 (402.67-467.5)	484.99 (416.08-561.09)	11.04%	8.19 (7.58-8.77)	5.69 (4.88-6.57)	-1.26 (-1.78 to -0.73)
Alaska	30.97 (28.62-33.07)	61.09 (52.32-70.73)	97.23%	9.74 (8.89-10.47)	6.01 (5.14-6.93)	-1.72 (-2.25 to -1.18)
Arizona	330.8 (302.13-352.25)	583.7 (496.36-685.71)	76.45%	7.07 (6.5-7.54)	4.74 (4.03-5.56)	-1.4 (-1.63 to -1.16)
Arkansas	258.44 (237.88-276.46)	262.86 (224.91-306.36)	1.71%	7.49 (6.93-7.99)	5.21 (4.45-6.09)	-1.2 (-1.33 to -1.07)
California	2498.07 (2310.49-2650.01)	3500.57 (2950.15-4057.56)	40.13%	7.58 (7.03-8.02)	5.53 (4.66-6.4)	-1.09 (-1.39 to -0.78)
Colorado	257.35 (237.19-275.77)	405.63 (331.33-484.3)	57.61%	7.19 (6.63-7.69)	4.35 (3.57-5.2)	-1.68 (-1.96 to -1.39)
Connecticut	416.67 (385.51-450.43)	333.14 (275.45-393.42)	-20.05%	9.12 (8.48-9.8)	4.98 (4.14-5.88)	-1.96 (-2.13 to -1.8)
Delaware	66.9 (61.85-71.7)	94.72 (80.22-109.59)	41.58%	8.01 (7.42-8.58)	5.18 (4.39-5.99)	-1.45 (-1.99 to -0.91)
District of Columbia	86.04 (79.29-92.36)	55.31 (46.34-65.58)	-35.72%	11.22 (10.39-12.06)	5.77 (4.88-6.84)	-2.11 (-2.44 to -1.77)
Florida	1573.82 (1432.36-1699.72)	1926.26 (1584-2320.94)	22.39%	7.29 (6.72-7.82)	4.69 (3.86-5.64)	-1.4 (-1.6 to -1.21)
Georgia	617.61 (576.77-660.37)	971.66 (818.11-1120.7)	57.33%	8.8 (8.21-9.41)	5.86 (4.94-6.77)	-1.39 (-1.7 to -1.07)
Hawaii	188.98 (174.25-204.13)	213.94 (177.31-253.65)	13.21%	14.57 (13.4-15.74)	7.88 (6.65-9.29)	-2.08 (-2.26 to -1.91)
Idaho	75.08 (68.3-81.07)	114.98 (95.55-135.3)	53.13%	6.03 (5.51-6.51)	3.82 (3.2-4.46)	-1.52 (-1.73 to -1.31)
Illinois	1246.08 (1144.57-1338.59)	1135.28 (942.16-1342.77)	-8.89%	8.42 (7.78-9)	5.28 (4.38-6.24)	-1.55 (-1.7 to -1.41)
Indiana	467.55 (436.01-499.7)	505.29 (424.35-588.87)	8.07%	6.5 (6.07-6.93)	4.52 (3.79-5.28)	-1.25 (-1.56 to -0.93)
Iowa	255.61 (229.35-280.53)	234.96 (192.51-278.26)	-8.08%	5.95 (5.38-6.49)	4.1 (3.37-4.86)	-1.26 (-2.03 to -0.5)
Kansas	223.92 (202.45-243.42)	219.56 (181.18-262.88)	-1.95%	6.48 (5.89-7.02)	4.51 (3.72-5.42)	-1.24 (-1.48 to -1)
Kentucky	383.84 (351.04-410.42)	422.52 (356.61-506.15)	10.08%	8.01 (7.36-8.55)	5.6 (4.74-6.66)	-1.09 (-1.66 to -0.51)
Louisiana	494.65 (456.69-528.73)	486.56 (412.12-576.78)	-1.63%	10.11 (9.36-10.79)	6.7 (5.67-7.93)	-1.29 (-1.51 to -1.06)
Maine	129.96 (118.45-140.93)	134.52 (112.39-158.99)	3.51%	7.66 (7.03-8.29)	4.73 (3.96-5.6)	-1.68 (-1.95 to -1.41)
Maryland	476.2 (439.62-513.3)	526 (436.95-622.85)	10.46%	8.61 (7.93-9.28)	5.07 (4.22-6)	-1.76 (-2.24 to -1.27)
Massachusetts	824.29 (736.36-903.73)	632.94 (513.71-746.58)	-23.21%	9.85 (8.89-10.72)	4.97 (4.07-5.87)	-2.14 (-2.63 to -1.66)
Michigan	932.01 (852.63-1005.25)	844.52 (701.24-999.3)	-9.39%	8.07 (7.39-8.69)	4.62 (3.85-5.47)	-1.82 (-1.89 to -1.74)
Minnesota	438.98 (397.55-473.86)	469.32 (379.46-561.89)	6.91%	7.61 (6.96-8.18)	4.69 (3.81-5.61)	-1.45 (-1.93 to -0.97)
Mississippi	269.82 (243.04-295.86)	289.77 (244.83-346.09)	7.4%	8.25 (7.48-9.03)	6.05 (5.09-7.22)	-1 (-1.64 to -0.35)
Missouri	475.87 (433-519.11)	502.87 (424.41-592.69)	5.68%	6.56 (6-7.13)	4.66 (3.93-5.48)	-1.29 (-1.64 to -0.93)
Montana	72.36 (65.56-78.48)	87.41 (72.67-104.48)	20.8%	6.61 (6.01-7.16)	4.21 (3.52-5.03)	-1.69 (-1.77 to -1.62)
Nebraska	152.29 (137.36-165.45)	142.23 (117.92-167.79)	-6.61%	6.67 (6.09-7.2)	4.31 (3.59-5.09)	-1.46 (-1.7 to -1.23)
Nevada	105.72 (97.47-113.74)	228.72 (191.56-268.98)	116.33%	7.73 (7.11-8.3)	4.58 (3.84-5.4)	-1.74 (-1.91 to -1.58)
New Hampshire	102.37 (94.19-109.9)	115.89 (96.82-138.27)	13.21%	7.64 (7.04-8.19)	4.32 (3.6-5.16)	-1.87 (-2.37 to -1.37)
New Jersey	1153.97 (1068.65-1226.33)	907.52 (748.12-1081.65)	-21.36%	10.96 (10.19-11.64)	5.6 (4.61-6.67)	-2.19 (-2.67 to -1.71)
New Mexico	142.14 (129.14-155.03)	219.62 (185.49-259.62)	54.52%	8.3 (7.56-9.02)	6.02 (5.09-7.09)	-1.09 (-1.33 to -0.86)
New York	2191.34 (2012.25-2344.67)	1943.41 (1592.92-2279.87)	-11.31%	9.1 (8.39-9.68)	5.54 (4.56-6.47)	-1.67 (-1.89 to -1.46)
North Carolina	605.65 (555.95-650.54)	857.33 (715.27-996.69)	41.56%	7.26 (6.68-7.78)	4.81 (4.02-5.6)	-1.42 (-1.5 to -1.34)
North Dakota	73.28 (65.91-79.94)	56.55 (46.59-66.75)	-22.83%	7.9 (7.18-8.57)	4.26 (3.54-4.98)	-1.93 (-2.46 to -1.39)
Ohio	1094.18 (1010.5-1171.38)	937.16 (792.78-1086.61)	-14.35%	7.56 (7.02-8.08)	4.46 (3.77-5.16)	-1.82 (-2.21 to -1.42)
Oklahoma	299.84 (273.99-323.2)	289.13 (246.72-338.17)	-3.57%	6.96 (6.4-7.48)	4.52 (3.86-5.3)	-1.42 (-1.73 to -1.1)
Oregon	262.75 (238.65-283.32)	329.09 (272.11-393.71)	25.25%	6.69 (6.11-7.23)	4.17 (3.47-5.01)	-1.57 (-1.79 to -1.34)
Pennsylvania	1634.66 (1499.24-1757.32)	1215.43 (1011.52-1427.17)	-25.65%	8.99 (8.29-9.63)	4.94 (4.11-5.79)	-1.96 (-2.15 to -1.78)
Rhode Island	149.05 (135.21-160.48)	110.44 (92.28-130.18)	-25.91%	10.06 (9.23-10.78)	5.35 (4.48-6.32)	-1.97 (-2.61 to -1.32)
South Carolina	324.65 (298.81-348.06)	490.34 (402.91-574.12)	51.03%	7.95 (7.34-8.52)	5.51 (4.55-6.45)	-1.25 (-1.78 to -0.71)
South Dakota	69.88 (62.85-76.83)	65.41 (55.52-76.05)	-6.4%	6.78 (6.17-7.44)	4.23 (3.58-4.91)	-1.54 (-1.73 to -1.36)
Tennessee	437.33 (407.35-469.2)	556.46 (472.48-650.58)	27.24%	6.86 (6.39-7.34)	4.77 (4.05-5.58)	-1.23 (-1.8 to -0.66)
Texas	1476.47 (1363.93-1579.33)	2283.03 (1950.51-2638.6)	54.63%	8.06 (7.46-8.61)	5.47 (4.68-6.31)	-1.32 (-1.73 to -0.92)
Utah	98.03 (89.75-104.93)	164.6 (138.92-191.68)	67.91%	6.1 (5.58-6.53)	4.03 (3.4-4.69)	-1.3 (-1.56 to -1.03)
Vermont	46.92 (42.99-50.24)	51.06 (43.75-59.55)	8.83%	6.67 (6.12-7.12)	3.96 (3.44-4.62)	-1.75 (-2.07 to -1.43)
Virginia	553.22 (515.67-585.95)	725.48 (605.81-845.11)	31.14%	7.79 (7.26-8.25)	5.02 (4.2-5.86)	-1.62 (-2.33 to -0.9)
Washington	440.64 (403.94-472.42)	562.85 (466.51-661.01)	27.74%	7.42 (6.84-7.92)	4.29 (3.56-5.04)	-1.72 (-1.81 to -1.63)
West Virginia	188.46 (174.39-201.15)	161.64 (137.17-189.42)	-14.23%	6.98 (6.48-7.44)	4.76 (4.02-5.59)	-1.46 (-2.03 to -0.88)
Wisconsin	546.59 (502.62-591.63)	502.66 (415.74-606.83)	-8.04%	8.11 (7.48-8.76)	4.72 (3.9-5.68)	-1.69 (-2.12 to -1.26)
Wyoming	32.52 (29.71-35.55)	41.81 (35.38-48.5)	28.56%	6.43 (5.88-7.01)	4.25 (3.62-4.91)	-1.26 (-1.63 to -0.89)

ASIR, age-standardized incidence rate; UI, uncertainty interval.

**Table 2:** Prevalence cases and ASPR of gastric cancer between 1990 and 2021 by gender and states.

Characteristics	Prevalence cases			ASPR per 100,000		
	1990 N (95% UI)	2021 N (95% UI)	Percentage change (%)	1990 N (95% UI)	2021 N (95% UI)	AAPC (95% CI))
**United States of America**	56133.24 (53081.02-57858.33)	74986.82 (70463.87-78357.48)	33.59%	17.92 (17.04-18.44)	13.88 (13.12-14.47)	-0.83 (-1.14 to -0.53)
Sex						
Female	20647.14 (18995.98-21605.59)	27280.79 (24831.13-28805.89)	32.13%	11.39 (10.64-11.85)	9.57 (8.87-10.04)	-0.53 (-0.86 to -0.2)
Male	35486.11 (34096.13-36528.43)	47706.03 (45068.29-49671.18)	34.44%	26.19 (25.17-26.95)	18.79 (17.8-19.56)	-1.11 (-1.44 to -0.78)
State						
Alabama	877.66 (806.96-951.43)	1118.21 (947.37-1303.54)	27.41%	16.81 (15.55-18.11)	13.62 (11.56-15.89)	-0.68 (-1.19 to -0.17)
Alaska	72.55 (66.14-78.07)	166.69 (142.27-193.43)	129.75%	20.43 (18.45-21.99)	16.28 (13.89-18.83)	-0.89 (-1.63 to -0.14)
Arizona	748.01 (683.53-812.73)	1567.08 (1319.16-1865.95)	109.5%	16.19 (14.95-17.54)	13.24 (11.2-15.61)	-0.82 (-0.9 to -0.74)
Arkansas	522.22 (478.62-567.32)	613.88 (514.73-723.41)	17.55%	15.76 (14.53-17.02)	12.73 (10.75-14.98)	-0.73 (-1.01 to -0.44)
California	5356.74 (4927.56-5710.02)	9434.15 (7890.34-10996.6)	76.12%	16.3 (15.01-17.4)	15.26 (12.88-17.76)	-0.25 (-0.55-0.06)
Colorado	595.86 (545.54-642.79)	1114.63 (913.59-1346.12)	87.06%	16.68 (15.29-18.03)	12.21 (10.07-14.71)	-0.98 (-1.19 to -0.77)
Connecticut	928.02 (860.82-1003.77)	890.15 (727.94-1052.12)	-4.08%	20.87 (19.41-22.56)	14.22 (11.74-16.78)	-1.31 (-1.95 to -0.67)
Delaware	142.06 (129.69-154.96)	245.32 (207.8-287.63)	72.68%	17.11 (15.63-18.62)	14.04 (11.92-16.47)	-0.66 (-1.3 to 0)
District of Columbia	140.42 (130.4-150.21)	126.77 (105.18-151.78)	-9.72%	18.77 (17.48-20.05)	13.56 (11.27-16.2)	-1.09 (-1.91 to -0.27)
Florida	3461.69 (3195.1-3747.22)	5092.62 (4202.61-6196.38)	47.11%	17.4 (16.2-18.72)	13.59 (11.29-16.46)	-0.78 (-1.24 to -0.32)
Georgia	1264.87 (1177.61-1368.5)	2598.23 (2192-3033.28)	105.41%	17.92 (16.72-19.38)	15.94 (13.47-18.56)	-0.44 (-0.6 to -0.29)
Hawaii	446.52 (410.71-485.03)	569.95 (473.93-673.49)	27.64%	34.13 (31.5-37.09)	22.45 (18.75-26.55)	-1.38 (-1.56 to -1.2)
Idaho	165.28 (149.72-180.69)	298.29 (244.88-348.96)	80.48%	13.54 (12.34-14.72)	10.21 (8.45-11.89)	-0.98 (-1.3 to -0.66)
Illinois	2604.67 (2388.33-2813.06)	3033.52 (2516.47-3641.21)	16.46%	18.02 (16.62-19.42)	14.66 (12.16-17.63)	-0.63 (-0.82 to -0.44)
Indiana	1026.9 (939.03-1115.46)	1266.31 (1046.73-1490.79)	23.31%	14.6 (13.35-15.77)	11.74 (9.74-13.77)	-0.75 (-1.1 to -0.39)
Iowa	602.93 (537.92-660.73)	635.16 (518.31-759.55)	5.35%	14.88 (13.39-16.26)	11.72 (9.55-13.92)	-0.76 (-1.13 to -0.39)
Kansas	509.16 (458.55-557.44)	570.23 (467.96-683.29)	11.99%	15.47 (14.01-16.84)	12.23 (10.02-14.65)	-0.82 (-1.19 to -0.45)
Kentucky	931.32 (846.37-1020.34)	1127.54 (946.57-1344.06)	21.07%	19.86 (18.09-21.71)	15.53 (13.12-18.42)	-0.81 (-1.36 to -0.26)
Louisiana	1117.1 (1031.57-1203.8)	1269.69 (1053.36-1511.51)	13.66%	23.06 (21.36-24.79)	17.93 (14.97-21.33)	-0.88 (-1.13 to -0.62)
Maine	295.48 (266.57-321.99)	345.87 (285.82-410.11)	17.05%	17.98 (16.33-19.53)	12.87 (10.63-15.3)	-1.24 (-1.52 to -0.96)
Maryland	1012.2 (926.46-1094.27)	1360.77 (1121.04-1628.55)	34.44%	18.25 (16.69-19.71)	13.59 (11.24-16.21)	-0.96 (-1.26 to -0.65)
Massachusetts	1923.02 (1726.49-2094.05)	1765.88 (1444.25-2106.09)	-8.17%	23.78 (21.6-25.86)	14.48 (11.81-17.24)	-1.62 (-2.24 to -0.99)
Michigan	2157.15 (1979.1-2334.91)	2257.2 (1856.32-2722.91)	4.64%	18.9 (17.41-20.4)	12.94 (10.69-15.53)	-1.18 (-1.31 to -1.05)
Minnesota	1063.28 (952.84-1161.09)	1313.43 (1076.57-1589.32)	23.53%	19.21 (17.42-20.79)	13.65 (11.23-16.51)	-1.03 (-1.91 to -0.14)
Mississippi	519.61 (470.82-572.5)	643.12 (532.14-773.52)	23.77%	16.39 (14.94-17.92)	13.9 (11.6-16.62)	-0.54 (-1.18-0.1)
Missouri	1011.54 (924.77-1109.18)	1261.69 (1061.73-1501.8)	24.73%	14.54 (13.36-15.82)	12.26 (10.32-14.51)	-0.56 (-1.29-0.18)
Montana	156.67 (141.37-171.7)	218.71 (184.44-263.33)	39.6%	14.69 (13.29-16.02)	11.06 (9.38-13.25)	-1.18 (-1.37 to -0.99)
Nebraska	340.05 (307.61-370.61)	376.06 (309.74-446.62)	10.59%	15.72 (14.35-17.05)	11.96 (9.87-14.16)	-0.97 (-1.08 to -0.87)
Nevada	230.43 (210.2-253.35)	595.2 (492.62-699.1)	158.3%	16.31 (14.91-17.88)	12.17 (10.11-14.3)	-0.9 (-1.05 to -0.75)
New Hampshire	236.69 (216.05-256.09)	312.96 (259.48-379.38)	32.23%	17.94 (16.38-19.39)	12.11 (10.01-14.63)	-1.3 (-1.8 to -0.8)
New Jersey	2731.61 (2512.74-2934.42)	2556.7 (2089.91-3056.25)	-6.4%	26.41 (24.33-28.19)	16.63 (13.62-19.85)	-1.49 (-2.02 to -0.96)
New Mexico	305.28 (279.95-332.81)	551.35 (462.19-650.65)	80.6%	17.87 (16.41-19.52)	16.02 (13.43-18.81)	-0.43 (-0.8 to -0.06)
New York	4539.24 (4183.76-4868.85)	5340.53 (4439.12-6270.73)	17.65%	19.34 (17.87-20.65)	15.95 (13.27-18.67)	-0.76 (-1.24 to -0.27)
North Carolina	1260.44 (1165.05-1357.14)	2167.18 (1818.66-2571.79)	71.94%	15.24 (14.1-16.36)	12.63 (10.62-15)	-0.6 (-1.13 to -0.06)
North Dakota	162.86 (145.69-180.5)	144.08 (118.31-170.24)	-11.53%	18.53 (16.74-20.4)	11.56 (9.53-13.69)	-1.52 (-2.11 to -0.93)
Ohio	2356.71 (2175.22-2554.47)	2287.28 (1906.44-2673.58)	-2.95%	16.61 (15.46-17.93)	11.4 (9.53-13.35)	-1.33 (-1.66 to -0.99)
Oklahoma	646.06 (591.22-705.27)	676.55 (572.24-807.92)	4.72%	15.52 (14.25-16.93)	11 (9.29-13.15)	-1.2 (-1.5 to -0.9)
Oregon	609.47 (555.13-666.92)	916.05 (759.96-1097.7)	50.3%	15.95 (14.58-17.55)	12.12 (10.05-14.55)	-0.85 (-1.51 to -0.19)
Pennsylvania	3516.01 (3198.46-3836.88)	3109.53 (2556.3-3705.02)	-11.56%	19.89 (18.27-21.66)	13.37 (11.11-15.83)	-1.29 (-1.45 to -1.14)
Rhode Island	327.7 (297.93-353.93)	292.82 (243.44-351.26)	-10.64%	22.96 (20.96-24.69)	14.87 (12.33-17.88)	-1.3 (-2.08 to -0.52)
South Carolina	649.29 (595.99-703.69)	1196.96 (982.03-1423.18)	84.35%	15.88 (14.59-17.18)	13.95 (11.49-16.47)	-0.53 (-1.18-0.11)
South Dakota	147.96 (133.11-164.33)	160.98 (136.3-191.05)	8.8%	15.27 (13.86-16.89)	11.11 (9.42-13.18)	-1.06 (-1.26 to -0.86)
Tennessee	902.47 (829.72-980.07)	1349.55 (1139.07-1575.02)	49.54%	14.42 (13.33-15.62)	11.97 (10.12-14.01)	-0.62 (-0.94 to -0.3)
Texas	3244.8 (2979.74-3526.35)	6117.09 (5200.16-7125.15)	88.52%	17.81 (16.37-19.31)	14.85 (12.65-17.27)	-0.62 (-1.06 to -0.18)
Utah	214.46 (195.65-231.54)	428.25 (358.37-504.55)	99.69%	13.32 (12.15-14.37)	10.58 (8.87-12.43)	-0.71 (-0.91 to -0.51)
Vermont	99.48 (90.37-108.37)	135.58 (115.03-159.8)	36.29%	14.53 (13.22-15.85)	11.06 (9.52-13.05)	-1.01 (-1.45 to -0.57)
Virginia	1232.34 (1144.7-1324.52)	1968.5 (1650.87-2343.74)	59.74%	17.34 (16.12-18.67)	14.04 (11.87-16.76)	-0.85 (-1.43 to -0.26)
Washington	1005.08 (924.38-1090.06)	1572.33 (1293.47-1858.04)	56.44%	17.24 (15.94-18.64)	12.43 (10.25-14.6)	-1 (-1.21 to -0.8)
West Virginia	385.48 (357.36-414.32)	373.66 (316.73-442.04)	-3.07%	14.7 (13.65-15.81)	11.69 (9.94-13.79)	-1.03 (-1.7 to -0.36)
Wisconsin	1265.51 (1151.29-1389.23)	1344.79 (1098.61-1642.51)	6.26%	19.5 (17.87-21.27)	13.18 (10.79-16.09)	-1.34 (-1.43 to -1.26)
Wyoming	70.9 (64.47-78.71)	107.74 (90.88-125.32)	51.97%	14.08 (12.8-15.61)	11.36 (9.68-13.15)	-0.62 (-1.1 to -0.14)

ASPR, age-standardized prevalence rate; UI, uncertainty interval.

**Table 3:** Death cases and ASMR of gastric cancer between 1990 and 2021 by gender and states.

Characteristics	Death cases			ASMR per 100,000		
	1990 N (95% UI)	2021 N (95% UI)	Percentage change (%)	1990 N (95% UI)	2021 N (95% UI)	AAPC (95% CI))
**United States of America**	17315.8 (16086.51-17976.84)	16444.97 (15029.59-17351.08)	-5.03%	5.36 (4.99-5.55)	2.84 (2.62-2.98)	-2.07 (-2.19 to -1.96)
Sex						
Female	7147.95 (6366.55-7541.19)	6679.85 (5857.21-7165.04)	-6.55%	3.63 (3.29-3.81)	2.07 (1.86-2.2)	-1.81 (-1.94 to -1.69)
Male	10167.85 (9707.73-10484.98)	9765.11 (9082.3-10213.68)	-3.96%	7.76 (7.37-8.02)	3.76 (3.5-3.93)	-2.36 (-2.47 to -2.24)
State						
Alabama	311.86 (287.47-334.22)	311.78 (267.84-361.33)	-0.03%	5.79 (5.35-6.18)	3.57 (3.08-4.14)	-1.57 (-2.11 to -1.03)
Alaska	19.82 (18.4-21.18)	33.79 (28.92-39.4)	70.48%	6.73 (6.16-7.26)	3.35 (2.86-3.89)	-2.41 (-2.67 to -2.15)
Arizona	217.26 (199.62-231.47)	331.52 (281.08-388.28)	52.59%	4.63 (4.25-4.92)	2.6 (2.21-3.04)	-1.97 (-2.18 to -1.76)
Arkansas	183.46 (169.19-195.83)	167.27 (142.78-194.2)	-8.82%	5.2 (4.82-5.54)	3.22 (2.75-3.74)	-1.53 (-1.67 to -1.4)
California	1804.79 (1667.25-1918.2)	2042.85 (1723.35-2352.43)	13.19%	5.48 (5.08-5.81)	3.16 (2.68-3.64)	-1.86 (-2.1 to -1.62)
Colorado	165.3 (151.83-176.39)	224.62 (183.92-267.67)	35.88%	4.62 (4.24-4.92)	2.37 (1.95-2.82)	-2.32 (-2.5 to -2.13)
Connecticut	278.22 (256.22-298.75)	192.25 (158.52-225.47)	-30.9%	5.98 (5.53-6.4)	2.7 (2.25-3.18)	-2.54 (-3.05 to -2.02)
Delaware	45.62 (41.96-48.8)	55.12 (47.15-63.5)	20.83%	5.46 (5.03-5.83)	2.9 (2.49-3.32)	-2.06 (-2.61 to -1.51)
District of Columbia	71.6 (66.01-76.69)	35.81 (29.62-42.67)	-49.99%	9.22 (8.52-9.87)	3.66 (3.04-4.36)	-2.97 (-3.28 to -2.66)
Florida	1072.67 (958.67-1151.88)	1132.18 (935.51-1336.84)	5.55%	4.79 (4.35-5.12)	2.59 (2.14-3.06)	-1.98 (-2.14 to -1.82)
Georgia	389.98 (362.74-415.27)	508.18 (430.29-583.2)	30.31%	5.58 (5.19-5.94)	3.02 (2.56-3.46)	-2.07 (-2.13 to -2.01)
Hawaii	122.02 (110.89-131.9)	125.04 (101.57-148)	2.48%	9.48 (8.6-10.24)	4.31 (3.58-5.12)	-2.74 (-2.82 to -2.66)
Idaho	50.28 (45.66-54.21)	67.17 (55.75-78.29)	33.61%	4 (3.64-4.3)	2.19 (1.83-2.54)	-2.02 (-2.13 to -1.9)
Illinois	863.54 (794.03-923.1)	647.56 (544.45-760.08)	-25.01%	5.76 (5.32-6.14)	2.91 (2.45-3.42)	-2.35 (-2.43 to -2.26)
Indiana	311.74 (288.14-331.54)	302.33 (254.4-351.22)	-3.02%	4.28 (3.97-4.55)	2.64 (2.22-3.06)	-1.63 (-1.88 to -1.38)
Iowa	157.99 (141.79-173.7)	131.03 (106.37-156.24)	-17.06%	3.52 (3.2-3.84)	2.17 (1.78-2.59)	-1.64 (-2.17 to -1.11)
Kansas	145.3 (131.85-156.31)	127.76 (106.12-151.2)	-12.07%	4.07 (3.71-4.37)	2.53 (2.1-2.99)	-1.51 (-1.75 to -1.27)
Kentucky	240.07 (219.89-255.56)	245.99 (206.73-293)	2.47%	4.94 (4.54-5.25)	3.17 (2.67-3.78)	-1.32 (-1.54 to -1.1)
Louisiana	314.74 (288.07-335.58)	275.27 (234.12-321.02)	-12.54%	6.39 (5.85-6.8)	3.69 (3.13-4.3)	-1.76 (-1.97 to -1.54)
Maine	84.23 (77.06-91)	78.43 (66.01-92.58)	-6.89%	4.86 (4.46-5.24)	2.64 (2.21-3.12)	-2.07 (-2.34 to -1.8)
Maryland	325.13 (299.84-350.46)	307.89 (254.71-361.81)	-5.3%	5.91 (5.44-6.37)	2.88 (2.37-3.37)	-2.3 (-2.54 to -2.05)
Massachusetts	524.76 (471.93-568.85)	345.42 (280.81-405.94)	-34.18%	6.13 (5.55-6.63)	2.61 (2.13-3.07)	-2.68 (-3.14 to -2.22)
Michigan	596.51 (542.92-642.86)	481.64 (404.19-564.32)	-19.26%	5.13 (4.68-5.52)	2.53 (2.12-2.96)	-2.32 (-2.39 to -2.24)
Minnesota	270.82 (241.92-292.44)	256.9 (210.78-305.15)	-5.14%	4.56 (4.14-4.9)	2.47 (2.03-2.95)	-1.98 (-2.37 to -1.59)
Mississippi	199.12 (180.51-217.43)	191.9 (162.66-227.92)	-3.63%	5.99 (5.47-6.52)	3.92 (3.32-4.66)	-1.35 (-2.09 to -0.6)
Missouri	324.78 (293.99-351.65)	300.1 (253.28-351.84)	-7.6%	4.36 (3.98-4.7)	2.68 (2.26-3.14)	-1.66 (-1.9 to -1.42)
Montana	48.66 (44.11-52.62)	52.42 (43.86-61.87)	7.71%	4.38 (3.98-4.71)	2.43 (2.05-2.87)	-2.05 (-2.21 to -1.89)
Nebraska	100.04 (89.64-108.21)	81.22 (66.8-95.82)	-18.81%	4.22 (3.83-4.56)	2.36 (1.97-2.79)	-1.85 (-2.05 to -1.65)
Nevada	70.68 (65.51-75.41)	133 (111.54-154.26)	88.17%	5.31 (4.89-5.67)	2.62 (2.2-3.05)	-2.27 (-2.53 to -2.01)
New Hampshire	65.3 (59.95-69.82)	64.73 (53.8-77.15)	-0.87%	4.83 (4.44-5.15)	2.34 (1.95-2.79)	-2.28 (-2.79 to -1.77)
New Jersey	711.19 (656.76-755.25)	488.9 (402.88-579.04)	-31.26%	6.68 (6.19-7.07)	2.87 (2.37-3.39)	-2.66 (-2.89 to -2.43)
New Mexico	98.68 (88.81-107.26)	134.68 (112.79-158.32)	36.48%	5.77 (5.21-6.26)	3.54 (2.96-4.16)	-1.71 (-1.88 to -1.53)
New York	1530.78 (1412.22-1632.75)	1081.78 (877.24-1269.86)	-29.33%	6.26 (5.8-6.64)	2.95 (2.43-3.46)	-2.47 (-3.12 to -1.81)
North Carolina	420.93 (387.56-451.88)	511.36 (430.43-590.47)	21.48%	5.04 (4.65-5.41)	2.79 (2.34-3.22)	-1.97 (-2.13 to -1.8)
North Dakota	48.55 (43.55-52.79)	33.57 (27.71-39.56)	-30.85%	5.05 (4.59-5.45)	2.4 (1.99-2.81)	-2.17 (-2.49 to -1.85)
Ohio	736.95 (679.78-787.08)	572.14 (484.56-663.68)	-22.36%	5.04 (4.66-5.37)	2.63 (2.22-3.04)	-2.19 (-2.65 to -1.72)
Oklahoma	203.01 (185.56-217.79)	182.65 (156.17-213.78)	-10.03%	4.62 (4.24-4.94)	2.78 (2.37-3.25)	-1.56 (-1.74 to -1.38)
Oregon	168.27 (153.72-181.74)	180.28 (148.53-214.24)	7.14%	4.22 (3.86-4.54)	2.2 (1.83-2.63)	-2.12 (-2.42 to -1.83)
Pennsylvania	1098.54 (1009.65-1175.46)	714.14 (599.99-838.81)	-34.99%	5.96 (5.48-6.37)	2.78 (2.32-3.26)	-2.45 (-2.61 to -2.3)
Rhode Island	98.83 (90-106.03)	63.15 (52.8-74.6)	-36.11%	6.52 (5.99-6.95)	2.93 (2.46-3.47)	-2.41 (-3 to -1.81)
South Carolina	233.95 (214.86-251.12)	301.76 (250.52-351.19)	28.99%	5.75 (5.29-6.16)	3.31 (2.77-3.84)	-1.86 (-1.94 to -1.77)
South Dakota	48.1 (43-52.27)	40.01 (33.33-46.55)	-16.84%	4.48 (4.06-4.84)	2.46 (2.07-2.86)	-1.94 (-2.08 to -1.8)
Tennessee	305.99 (282.36-327.19)	341.24 (291.65-398.38)	11.52%	4.75 (4.4-5.08)	2.85 (2.45-3.34)	-1.7 (-2.35 to -1.04)
Texas	990.81 (909.9-1060.88)	1302.29 (1115.39-1490.01)	31.44%	5.39 (4.95-5.75)	3.09 (2.64-3.53)	-1.83 (-2.26 to -1.39)
Utah	65.52 (59.68-69.94)	94.21 (79.1-109.39)	43.78%	4.09 (3.72-4.36)	2.29 (1.93-2.66)	-1.8 (-2.03 to -1.57)
Vermont	31.89 (29.11-34.16)	29.04 (25.05-33.58)	-8.96%	4.46 (4.09-4.77)	2.16 (1.88-2.5)	-2.43 (-2.8 to -2.06)
Virginia	364.63 (338.5-385.5)	407.15 (340.42-477.17)	11.66%	5.15 (4.77-5.43)	2.75 (2.32-3.23)	-2.2 (-2.73 to -1.67)
Washington	280.39 (255.3-301.36)	303.53 (254.37-358.18)	8.25%	4.67 (4.28-4.99)	2.24 (1.88-2.63)	-2.35 (-2.53 to -2.18)
West Virginia	131.84 (121.96-141.3)	102.42 (86.81-120.71)	-22.32%	4.81 (4.47-5.15)	2.89 (2.45-3.4)	-1.87 (-2.26 to -1.49)
Wisconsin	348.78 (315.79-374.24)	285.05 (235.85-342.94)	-18.27%	5.05 (4.61-5.41)	2.58 (2.13-3.1)	-2.24 (-2.39 to -2.08)
Wyoming	21.87 (19.91-23.83)	24.47 (20.59-28.26)	11.89%	4.32 (3.94-4.7)	2.42 (2.05-2.78)	-1.76 (-2.05 to -1.47)

ASMR, age-standardized mortality rates; UI, uncertainty interval.

**Table 4:** DALYs and ASDR of gastric cancer between 1990 and 2021 by gender and states.

Characteristics	DALYs			ASDR per 100,000		
	1990 N (95% UI)	2021 N (95% UI)	Percentage change (%)	1990 N (95% UI)	2021 N (95% UI)	AAPC (95% CI))
**United States of America**	383156.97 (366566.62-393934.5)	363139.06 (342100.1-377924.87)	-5.22%	124.9 (120.01-128.28)	69.16 (65.84-71.77)	-1.91 (-1.99 to -1.83)
Sex						
Female	143814.14 (133644.79-149493.35)	140111.18 (128747.3-147535.3)	-2.57%	81.68 (77.09-84.4)	50.9 (47.65-53.33)	-1.49 (-1.65 to -1.33)
Male	239342.83 (231729.75-245903.27)	223027.88 (212329.17-231303.12)	-6.82%	179.27 (173.54-184.34)	89.92 (85.76-93.21)	-2.25 (-2.33 to -2.17)
State						
Alabama	7035.76 (6595.51-7451.1)	7089.84 (6075.6-8240.91)	0.77%	137.57 (129.42-145.22)	89.06 (76.22-103.28)	-1.47 (-2.11 to -0.82)
Alaska	567.78 (530.02-604.08)	823.31 (716.13-954.91)	45%	154.64 (143.52-165.39)	82.33 (71.72-95.1)	-2.17 (-2.42 to -1.92)
Arizona	4878.02 (4539.02-5181.95)	7313.02 (6226.21-8576.26)	49.92%	109.08 (101.98-115.76)	64.31 (54.57-75.06)	-1.81 (-2 to -1.61)
Arkansas	3980.55 (3723.24-4224.8)	3797.51 (3230.3-4416.42)	-4.6%	123.66 (115.87-131.2)	81.3 (69.12-94.83)	-1.31 (-1.45 to -1.17)
California	41485.81 (39119.69-43607.18)	46729.3 (39506.48-53722.36)	12.64%	128.64 (121.29-135.25)	78.98 (66.99-90.83)	-1.66 (-1.92 to -1.41)
Colorado	3748.15 (3514.38-3985.82)	4944.57 (4075.34-5917.9)	31.92%	105.7 (98.98-112.37)	55.66 (45.9-66.27)	-2.17 (-2.34 to -1.99)
Connecticut	5955.82 (5575.39-6370.14)	4010.81 (3336.62-4754.49)	-32.66%	136.24 (128.08-145.37)	64.47 (53.19-76.17)	-2.35 (-3.09 to -1.6)
Delaware	1033.02 (966.52-1098.22)	1195.64 (1031.49-1376.65)	15.74%	126.54 (118.58-134.36)	71.11 (61.6-81.64)	-1.84 (-2.31 to -1.37)
District of Columbia	1784.47 (1658.89-1903.62)	851.42 (720.97-1011.49)	-52.29%	242.22 (224.99-258.71)	91.58 (77.34-108.83)	-3.06 (-3.42 to -2.69)
Florida	22665.57 (20999.56-24121.04)	24678.17 (20488.96-29294.83)	8.88%	113.89 (106.62-120.4)	65.38 (54.32-77.35)	-1.71 (-1.91 to -1.5)
Georgia	9368.61 (8828.37-9926.57)	11699.08 (9972.02-13554.41)	24.88%	134.43 (126.55-142.34)	72.92 (62.2-84.6)	-1.96 (-2.08 to -1.84)
Hawaii	2761.29 (2573.8-2955.58)	2619.9 (2208.54-3111.9)	-5.12%	214.63 (200.08-229.19)	106 (89.35-126.37)	-2.24 (-2.61 to -1.86)
Idaho	1070.98 (993.47-1138.14)	1406.98 (1175.61-1645.42)	31.37%	89.88 (83.73-95.52)	49.93 (42.12-58.06)	-1.83 (-2.07 to -1.59)
Illinois	18880.28 (17727.61-19965.75)	14223.02 (11908.32-16744.94)	-24.67%	133.18 (125.79-140.53)	70.36 (59.09-82.81)	-2.21 (-2.29 to -2.12)
Indiana	6843.3 (6409.33-7264.82)	6774.01 (5689.05-7908.14)	-1.01%	99.1 (93.07-105.08)	64.56 (54.22-75.38)	-1.31 (-1.48 to -1.14)
Iowa	3165.34 (2899.63-3431.79)	2727.06 (2240.05-3259.67)	-13.85%	80.07 (73.91-86.41)	51.52 (42.26-61.36)	-1.35 (-2.05 to -0.65)
Kansas	3034.42 (2789.23-3241.08)	2743.31 (2285.24-3242.89)	-9.59%	94.7 (87.9-100.85)	60.43 (50.18-71.71)	-1.4 (-1.6 to -1.21)
Kentucky	5371.46 (5020.91-5685.9)	5551.49 (4689.85-6674.19)	3.35%	116.38 (109.03-123.22)	78.16 (66.04-93.42)	-1.19 (-1.37 to -1.01)
Louisiana	7272.03 (6787.9-7703.82)	6430.85 (5464.97-7561.28)	-11.57%	152.39 (142.76-160.79)	92.79 (78.76-109.36)	-1.58 (-1.79 to -1.37)
Maine	1764.16 (1629.11-1895)	1629.97 (1359.19-1930.68)	-7.61%	109.6 (101.74-117.28)	63.2 (52.74-74.8)	-1.82 (-2.09 to -1.54)
Maryland	7486.13 (6963.06-8015.52)	6864.19 (5689.71-8147.28)	-8.31%	136.21 (126.46-145.75)	70.1 (58.24-83.25)	-2.06 (-2.32 to -1.8)
Massachusetts	10884.53 (10001.14-11702.27)	7061.66 (5772.09-8257.72)	-35.12%	138 (127.73-147.4)	59.26 (48.72-69.21)	-2.7 (-3.26 to -2.15)
Michigan	13289.89 (12318-14212.91)	10391.85 (8778.32-12188.38)	-21.81%	118.36 (109.95-126.18)	61 (51.59-71.45)	-2.06 (-2.21 to -1.91)
Minnesota	5489.57 (5057.68-5887.3)	5228 (4324.31-6274.17)	-4.76%	101.32 (94.34-108.31)	55.64 (45.94-66.54)	-2.05 (-2.29 to -1.81)
Mississippi	4431.67 (4076.66-4817.71)	4447.02 (3737.58-5296.05)	0.35%	142.71 (131.65-154.6)	98.88 (83.42-117.69)	-1.34 (-1.88 to -0.79)
Missouri	6889.72 (6327.17-7402.32)	6539.37 (5506.07-7662.79)	-5.09%	101.45 (93.5-108.83)	65.39 (55.01-76.91)	-1.58 (-1.76 to -1.39)
Montana	1030.81 (947.41-1108.58)	1092.22 (915.39-1304.36)	5.96%	98.98 (91.17-106.7)	57.74 (48.77-68.54)	-1.86 (-2.02 to -1.7)
Nebraska	2020.41 (1856.93-2174.45)	1689.99 (1409.16-2003.44)	-16.35%	95.81 (88.69-102.77)	55.13 (45.79-65.48)	-1.68 (-1.93 to -1.44)
Nevada	1722.4 (1609.47-1831.75)	3015.38 (2517.46-3524.71)	75.07%	123.38 (115.33-131.1)	63.13 (52.67-73.77)	-2.16 (-2.47 to -1.84)
New Hampshire	1402.3 (1295.76-1488.22)	1327.17 (1102.3-1592.44)	-5.36%	107.85 (99.82-114.49)	52.97 (44.38-63.4)	-2.22 (-2.72 to -1.73)
New Jersey	15601.07 (14636.51-16444.97)	10265.88 (8435.98-12162.45)	-34.2%	152.91 (144.14-161.09)	66.94 (55.03-79.53)	-2.65 (-3.09 to -2.2)
New Mexico	2306.81 (2124.16-2497.16)	3068.58 (2578.5-3604.78)	33.02%	137.24 (126.72-148.21)	92.65 (77.82-108.62)	-1.31 (-1.48 to -1.14)
New York	34112.99 (32003.04-36034.36)	23400.83 (19473.68-27398.78)	-31.4%	148.15 (140.33-156.16)	71.69 (60.23-83.68)	-2.33 (-2.87 to -1.78)
North Carolina	9653.83 (8992.15-10250.39)	11278.59 (9420.62-13092.28)	16.83%	118.69 (110.63-126)	67.78 (57.1-79.19)	-1.75 (-1.92 to -1.57)
North Dakota	968.19 (885.06-1044.29)	704.27 (586.67-825.18)	-27.26%	113.85 (105.27-122.18)	58.12 (48.64-68.04)	-2.17 (-2.65 to -1.68)
Ohio	16028.13 (15122.03-17017.17)	12233.24 (10323.47-14198.58)	-23.68%	115.19 (109.06-121.89)	62.9 (52.94-73.07)	-2.09 (-2.48 to -1.7)
Oklahoma	4429.88 (4119.99-4750.89)	4101.9 (3506.39-4799.72)	-7.4%	109 (101.65-116.67)	68.6 (58.75-80.38)	-1.41 (-1.86 to -0.95)
Oregon	3579.85 (3324.07-3844.51)	3866.26 (3211.83-4632.11)	8%	96.19 (89.69-103.26)	53.08 (44.01-63.63)	-2.06 (-2.51 to -1.61)
Pennsylvania	23272.69 (21641.8-24866.13)	14798.47 (12342.92-17330.09)	-36.41%	135.66 (126.44-144.71)	65.52 (54.42-76.75)	-2.39 (-2.84 to -1.95)
Rhode Island	2071.97 (1926.03-2196.11)	1274.07 (1071.28-1520.81)	-38.51%	149.73 (139.66-158.36)	66.02 (55.51-78.89)	-2.51 (-3.17 to -1.85)
South Carolina	5616.87 (5210.29-5993.83)	6870.96 (5721.88-8054.22)	22.33%	139.45 (129.61-148.38)	83.25 (69.21-98.3)	-1.72 (-1.81 to -1.64)
South Dakota	960.97 (877.06-1040.23)	840.54 (710.38-973.12)	-12.53%	102.15 (94.2-110.48)	59.94 (51.44-69.07)	-1.72 (-1.85 to -1.58)
Tennessee	6908.39 (6506.32-7350.44)	7802.76 (6668.66-9169.52)	12.95%	112.4 (105.58-119.44)	71.46 (60.77-83.93)	-1.42 (-1.57 to -1.27)
Texas	23204.82 (21689.75-24614.24)	30849.34 (26385.43-35370.81)	32.94%	128.97 (120.56-136.62)	76.3 (65.4-87.25)	-1.72 (-2.14 to -1.3)
Utah	1431.42 (1329.24-1526.97)	2050.71 (1729.55-2376.44)	43.26%	90.58 (84.32-96.53)	51.56 (43.53-59.74)	-1.87 (-2.3 to -1.44)
Vermont	677.93 (626.64-721.96)	601.45 (523.9-699.45)	-11.28%	100.37 (92.89-106.8)	51.09 (44.65-59.36)	-2.17 (-2.5 to -1.83)
Virginia	8392.53 (7920.36-8818.08)	8921.41 (7498.05-10468.19)	6.3%	119.35 (112.61-125.5)	65.28 (54.97-76.54)	-2.13 (-2.76 to -1.49)
Washington	6174.3 (5741.62-6557.2)	6562.52 (5479.25-7685.26)	6.29%	107 (100.02-113.75)	52.68 (43.84-61.48)	-2.19 (-2.37 to -2.01)
West Virginia	2843.88 (2671.77-3022.14)	2270.38 (1919.12-2685.83)	-20.17%	111.1 (104.67-117.61)	73.97 (62.65-87.21)	-1.46 (-1.75 to -1.17)
Wisconsin	7122.41 (6592.97-7579.77)	5949.29 (4946.99-7180.22)	-16.47%	112.53 (104.7-119.72)	59.98 (49.83-72.22)	-2.04 (-2.19 to -1.89)
Wyoming	483.8 (445.63-524.52)	531.51 (456.5-615.34)	9.86%	97.15 (89.64-105.4)	58.04 (49.97-66.6)	-1.65 (-1.89 to -1.41)

DALYs, disability-adjusted life years; ASDR, age-standardized DALYs rates; UI, uncertainty interval.

### GC burden by age and sex in the USA

**Figure 2:**
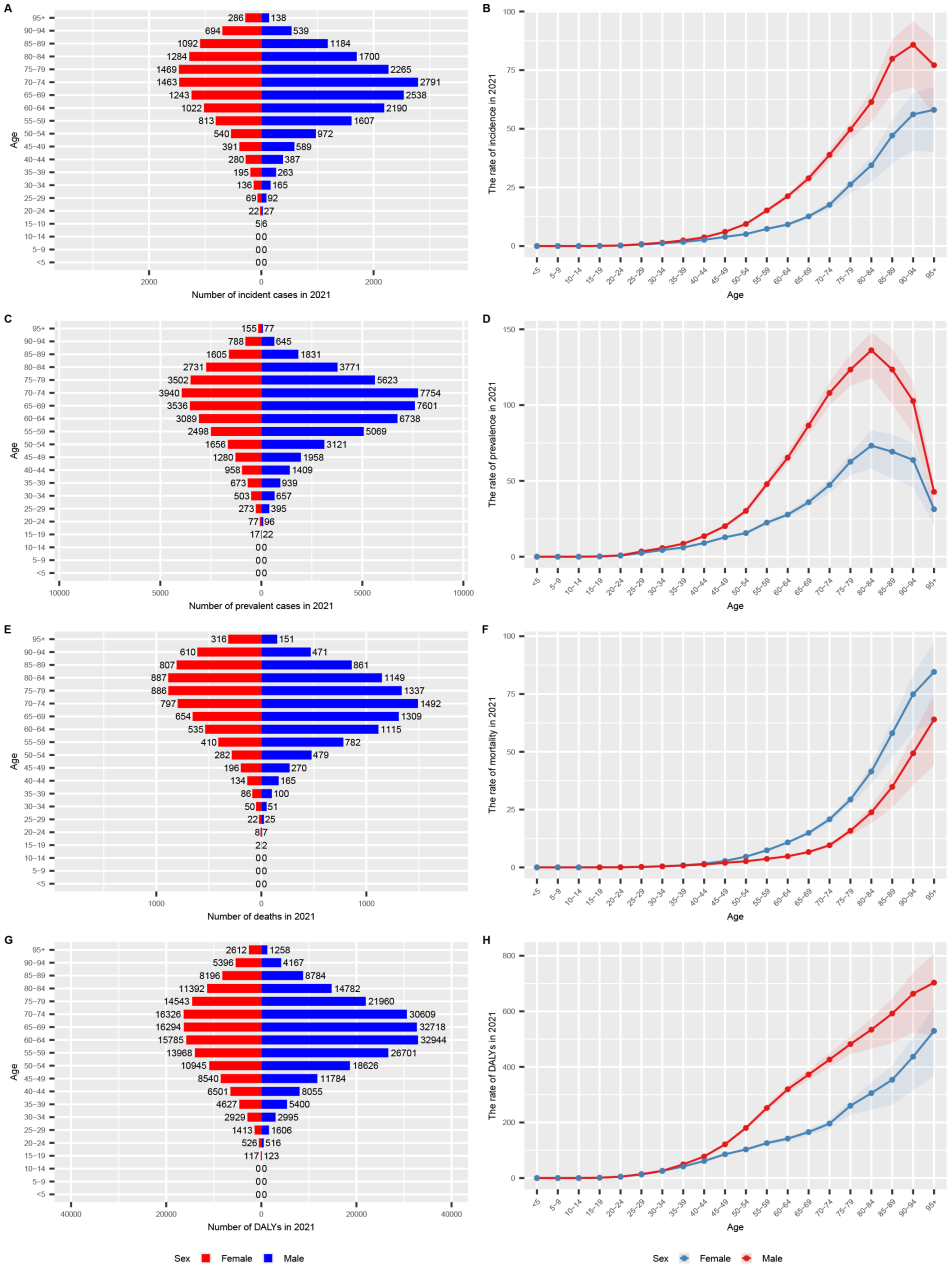
Age-specific numbers and rates of GC burden by sex and subtype in 2021 in the USA. **(A)** age-specific numbers and rates of incidence (bar chart); **(B)** age-specific numbers and rates of incidence (line chart); **(C)** age-specific numbers and rates of prevalence (bar chart); **(D)** age-specific numbers and rates of prevalence (line chart); **(E)** age-specific numbers and rates of deaths (bar chart); **(F)** age-specific numbers and rates of deaths (line chart); **(G)** age-specific numbers and rates of DALYs (bar chart); **(H)** age-specific numbers and rates of DALYs (line chart). DALYs, disability-adjusted life years; UI, uncertainty interval; GC, gastric cancer.

In 2021, the incidence and prevalence of GC among men in the USA were higher than those among women, a trend observed across all age groups (**Tables 1**, **2**; **Figures 2A-D**). Specifically, the highest number of incident and prevalent cases among women occurred in the 75-79 age group, whereas among men, the peak was in the 70-74 age group. In terms of incidence rates, a rising trend was observed in men up to the 90-94 age group, followed by a decline, while in women, the incidence rate consistently increased with age. Regarding prevalence rates, both genders exhibited an increasing trend up to the 80-84 age group, after which the rates declined. The study further revealed that mortality rates were higher among women across all age groups (**Figures 2E, F**). As for DALYs, the analysis showed that men experienced a higher burden across all age groups compared to women (**Figures 2G, H**). Both mortality rates and DALYs demonstrated a general upward trend with increasing age, indicating a significant burden of GC among elderly patients.

### Age-Period-Cohort model estimation of GC trends in the USA

**Figure 3:**
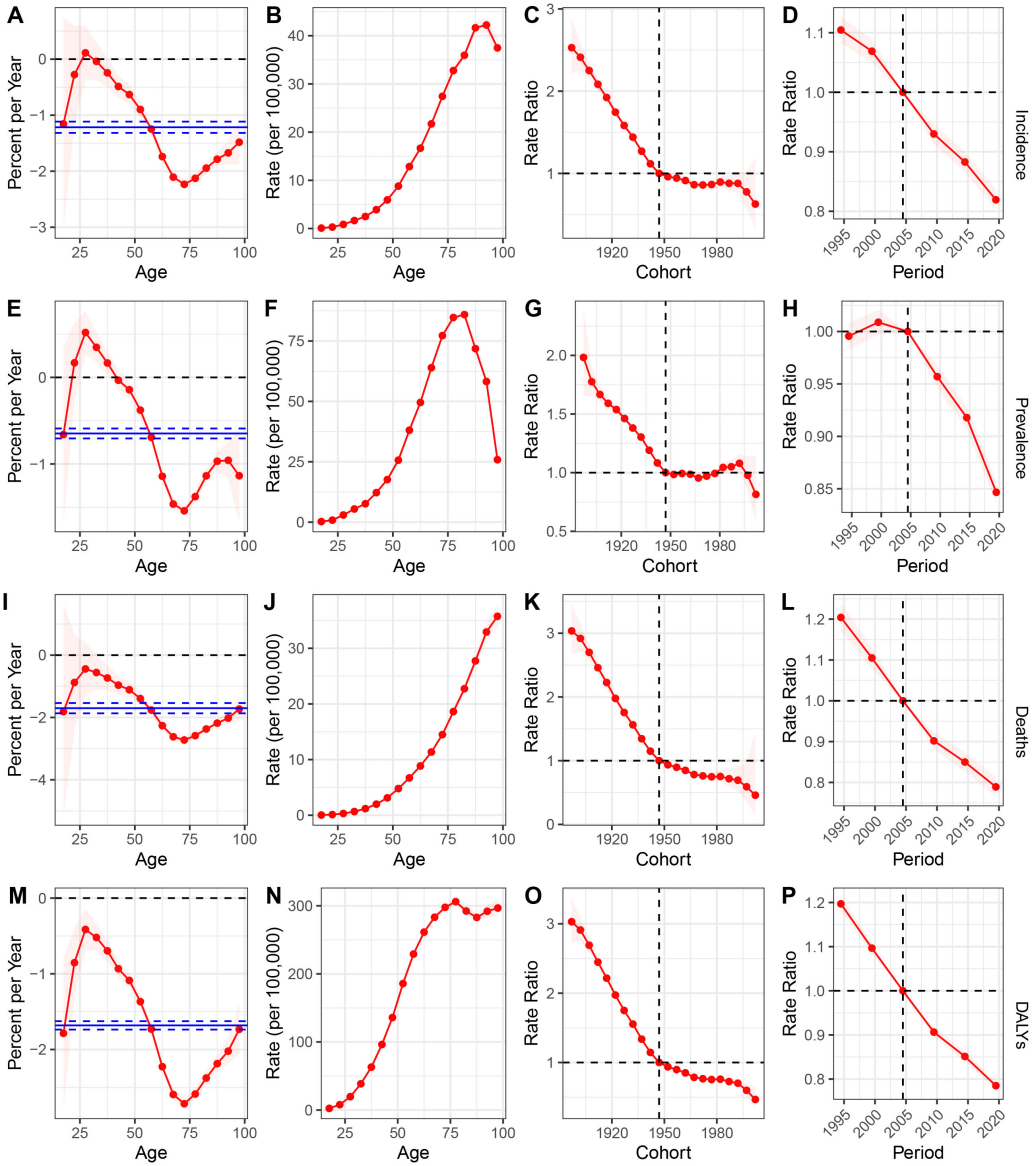
**(A-D)** incidence: **(A)** Local drift with net drift values in USA from 1990 to 2021; **(B)** Fitted longitudinal age curves; **(C)** The rate ratio of each period compared with the reference adjusted for age and nonlinear cohort effects; **(D)** The rate ratio of each cohort compared with the reference adjusted for age and nonlinear period effects; **(E–H)**, prevalence: **(E)** Local drift with net drift values in USA from 1990 to 2021.; **(F)** Fitted longitudinal age curves; **(G)** The rate ratio of each period compared with the reference adjusted for age and nonlinear cohort effects; **(H)** The rate ratio of each cohort compared with the reference adjusted for age and nonlinear period effects; **(I)** Local drift with net drift values in USA from 1990 to 2021.; **(J)** Fitted longitudinal age curves; **(K)** The rate ratio of each period compared with the reference adjusted for age and nonlinear cohort effects; **(L)** The rate ratio of each cohort compared with the reference adjusted for age and nonlinear period effects; **(M)** Local drift with net drift values in USA from 1990 to 2021.; **(N)** Fitted longitudinal age curves; **(O)** The rate ratio of each period compared with the reference adjusted for age and nonlinear cohort effects; **(P)** The rate ratio of each cohort compared with the reference adjusted for age and nonlinear period effects. DALYs, disability-adjusted life years; GC, gastric cancer.

We utilized the Age-Period-Cohort model to estimate the age, period, and cohort effects on the incidence, prevalence, mortality, and DALYs for GC in the USA (**Figure 3**). The age effect is illustrated through longitudinal age curves, depicting the natural history of GC incidence. The period effect reflects the relative risk of incidence over time, enabling tracking of temporal changes in incidence rates. Additionally, the cohort effect shows the relative risk of incidence across different birth cohorts, allowing observation of variations in incidence among different birth groups. During the study period, the net drift percentages per year for GC incidence, prevalence, mortality, and DALYs were -1.22 (95% CI: -1.32 to -1.12), -0.65 (95% CI: -0.71 to -0.59), -1.70 (95% CI: -1.87 to -1.54), and -1.68 (95% CI: -1.74 to -1.63), respectively, indicating significant reductions in GC incidence, prevalence, mortality, and DALYs (**Figures 3A, E, I, M**). The local drift trends for incidence, prevalence, mortality, and DALYs initially increased, then decreased, and subsequently increased again. The age effect revealed that both incidence and prevalence rates rise gradually from infancy, peak around 90 years of age, and then decline annually. In terms of mortality and DALYs, we observed a continuous increase with advancing age (**Figures 3B, F, J, N**). The cohort effect indicated a downward trend in GC incidence, prevalence, mortality, and DALYs for individuals born across all periods (**Figures 3C, G, K, O**). The period effect generally showed a persistent decline in incidence, mortality, and DALYs, while prevalence exhibited a fluctuating trend, with an increase in 2000 followed by a subsequent decline (**Figures 3D, H, L, P**).

### Decomposition analysis of GC trends in the USA

**Figure 4:**
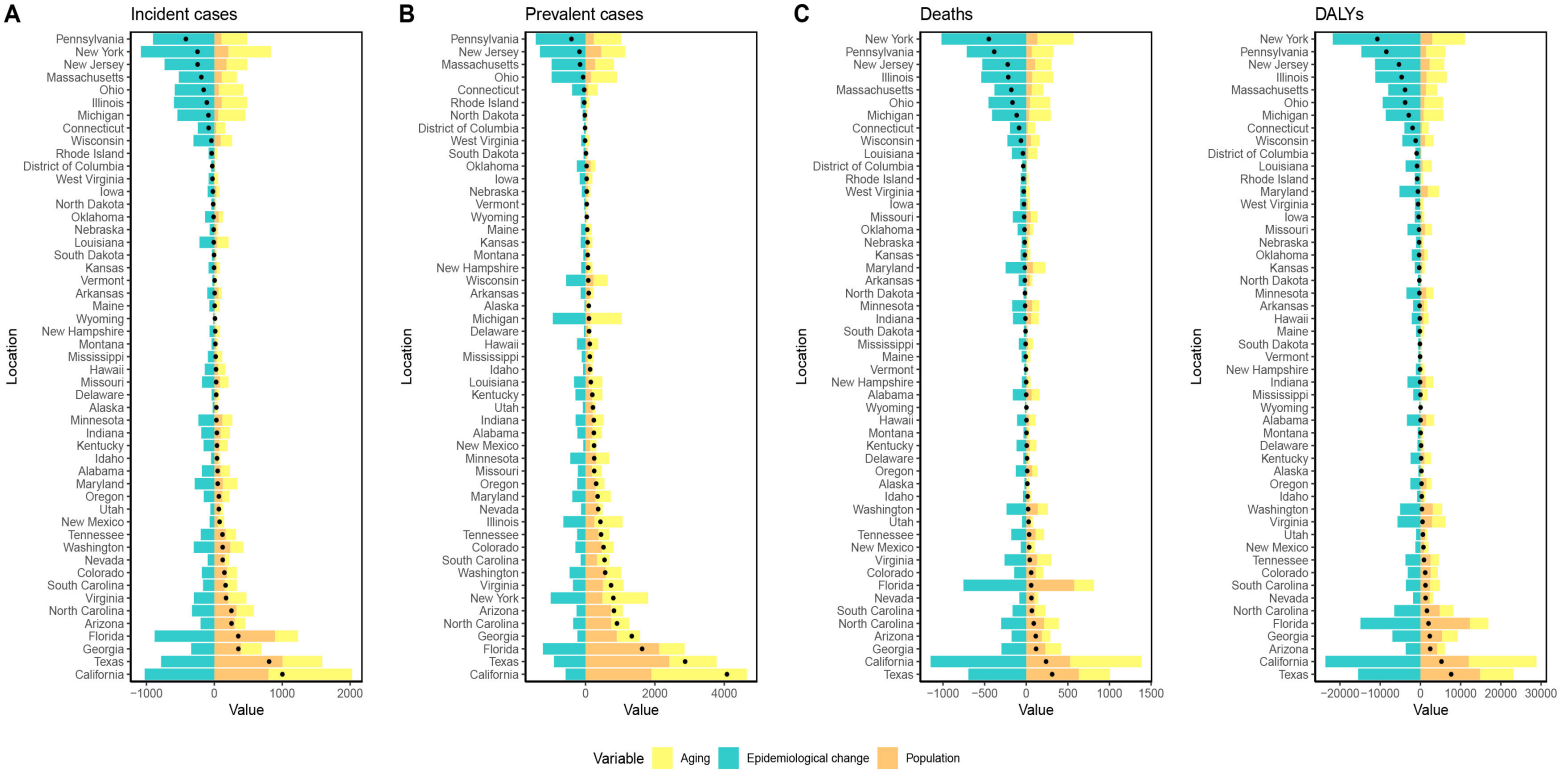
Visualization of decomposition analysis results. **(A)** decomposition analysis for ASIR; **(B)** decomposition analysis for ASPR; **(C)** decomposition analysis for ASMR; **(D)** decomposition analysis for ASDR. Black dots represent the overall changes in disease burden due to aging, epidemiological changes, and population growth. For each component, an increase in the disease burden of GC related to that component is indicated by positive values, whereas a decrease is indicated by negative values; ASIR, age-standardized incidence rate; ASPR, age-standardized prevalence rate; ASMR, age-standardized mortality rate; ASDR, age-standardized DALYs rate. DALYs, disability-adjusted life years; GC, gastric cancer.

Decomposition analyses of the number of new cases of GC, the number of prevalent cases, the number of deaths, and DALYs across each state in the USA revealed that population aging had a positive impact on the GC burden in all states. Population growth contributed positively to the rise in new cases, prevalent cases, deaths, and DALYs in all states except West Virginia. In contrast, changes in epidemiologic trends contributed negatively to the increase in new GC cases, prevalent cases, deaths, and DALYs across all states (**Figure 4**, **Supplementary Tables S5**-**S8**).

### Forecast of GC trends in the USA for the next 15 years

**Figure 5:**
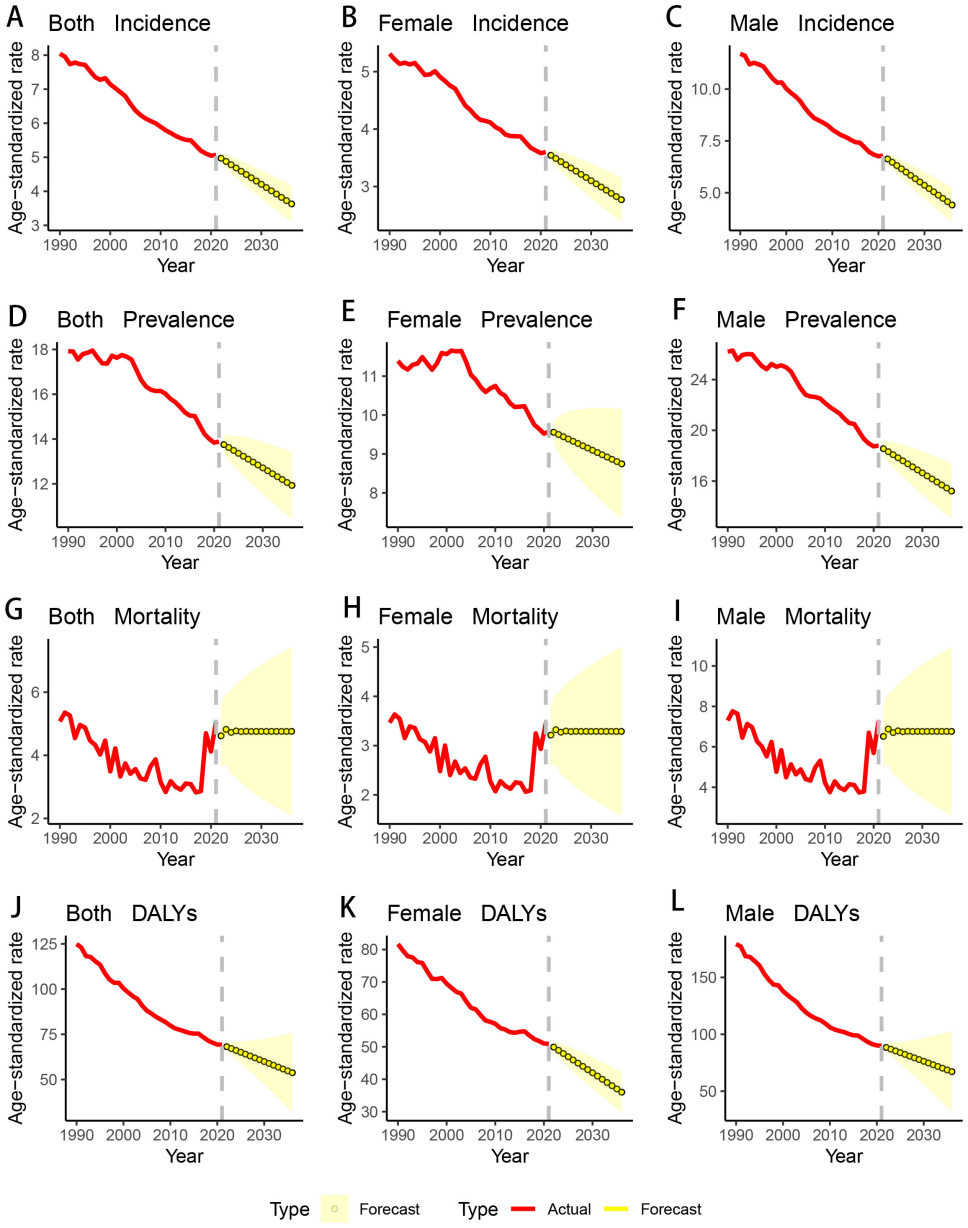
Changing trend and prediction rate of GC burden from 2022 to 2035 in the USA. **(A)** ASIR from 1990 to 2036; **(B)** ASIR from 1990 to 2036 for female; **(C)** ASIR from 1990 to 2036 for male; **(D)** ASPR from 1990 to 2036; **(E)** ASPR from 1990 to 2036 for female; **(F)** ASPR from 1990 to 2036 for male; **(G)** ASMR from 1990 to 2036; **(H)** ASMR from 1990 to 2036 for female; **(I)** ASMR from 1990 to 2036 for male; **(J)** ASDR from 1990 to 2036; **(K)** ASDR from 1990 to 2036 for female; **(L)** ASDR from 1990 to 2036 for male; ASIR: age-standardized incidence rate; ASPR: age-standardized prevalence rate; ASMR: age-standardized mortality rate; ASDR: age-standardized DALYs rate. DALYs: disability-adjusted life years; GC: gastric cancer.

Using the ARIMA model, we forecasted the trends of ASIR, ASPR, ASMR, and ASDR for GC from 2022 to 2036, by gender. The projections indicate that the ASIR, ASPR, and ASDR are expected to continue their overall decline, with both men and women showing an upward trend. Conversely, the ASMR is anticipated to rise over the next 15 years (**Table 5**; **Figure 5**).

**Table 5:** Detailed results of the decomposition analysis for gastric cancer.

Year	ASIR			ASPR			ASMR			ASDR		
	Both	Female	Male	Both	Female	Male	Both	Female	Male	Both	Female	Male
2022	4.97	3.54	6.63	13.75	9.56	18.55	4.62	3.21	6.51	68.14	49.91	88.41
2023	4.88	3.49	6.47	13.62	9.50	18.32	4.82	3.32	6.88	67.11	48.92	86.89
2024	4.78	3.43	6.31	13.49	9.44	18.08	4.73	3.27	6.70	66.09	47.92	85.37
2025	4.68	3.38	6.15	13.36	9.38	17.84	4.77	3.30	6.79	65.06	46.93	83.85
2026	4.59	3.32	5.99	13.23	9.33	17.60	4.75	3.28	6.75	64.03	45.94	82.33
2027	4.49	3.27	5.84	13.10	9.27	17.36	4.76	3.29	6.77	63.01	44.95	80.82
2028	4.40	3.21	5.68	12.97	9.21	17.12	4.76	3.29	6.76	61.98	43.95	79.30
2029	4.30	3.16	5.52	12.84	9.15	16.88	4.76	3.29	6.76	60.96	42.96	77.78
2030	4.20	3.10	5.36	12.71	9.09	16.65	4.76	3.29	6.76	59.93	41.97	76.26
2031	4.11	3.05	5.20	12.58	9.04	16.41	4.76	3.29	6.76	58.91	40.97	74.74
2032	4.01	2.99	5.04	12.45	8.98	16.17	4.76	3.29	6.76	57.88	39.98	73.23
2033	3.92	2.94	4.88	12.32	8.92	15.93	4.76	3.29	6.76	56.86	38.99	71.71
2034	3.82	2.88	4.73	12.19	8.86	15.69	4.76	3.29	6.76	55.83	38.00	70.19
2035	3.73	2.83	4.57	12.06	8.80	15.45	4.76	3.29	6.76	54.80	37.00	68.67
2036	3.63	2.77	4.41	11.93	8.75	15.21	4.76	3.29	6.76	53.78	36.01	67.15

ASIR, age-standardized incidence rate; ASPR, age-standardized prevalence rates; ASMR, age-standardized mortality rates; ASDR, age-standardized DALYs rates; DALYs, disability-adjusted life years.

## Discussion

GC remains a significant cause of morbidity and mortality in many regions worldwide, with the global incidence and death toll continuing to rise (23). In this study, we estimated the incidence, prevalence, mortality, and DALYs of GC in the USA from 1990 to 2021. Our findings indicate that, on a national scale, the absolute numbers of GC cases and prevalence have steadily increased, while the number of deaths and GC-related DALYs have shown a decline. Despite the increase in absolute numbers, we observed a consistent downward trend in the ASIR, ASPR, ASMR, and ASDR over the past 30 years (AAPC < 0). We also found that the disease burden of GC is higher in males compared to females, and that it predominantly affects patients aged 60 and above. Additionally, using the ARIMA model, we projected the trend of GC disease burden in the USA over the next 15 years. Our predictions suggest that the overall disease burden will continue to decline, with the exception of ASMR, which may experience a slight increase.

From 1990 to 2021, there has been a significant decline in the ASIR, ASPR, ASMR, and ASDR for GC in the USA. This trend not only falls markedly below the global average but also aligns closely with findings reported in other relevant studies (2). This sustained decline is likely attributable to significant advancements in GC diagnosis and treatment management in the USA. Firstly, the widespread adoption of gastroscopy in the USA has been pivotal, as it enables early detection of many GC cases, facilitates timely treatment, and consequently contributes to a significant reduction in patient mortality rates (24). Additionally, as one of the most advanced countries in terms of medical technology, the USA has remained at the forefront of GC research and treatment. The American medical community has conducted extensive research into the pathological mechanisms and treatment strategies for GC, and has been able to rapidly implement the latest research findings into clinical practice. This includes the application of frontier therapies such as immunotherapy, targeted therapy, and neoadjuvant chemoradiotherapy, which have significantly improved the overall survival and progression-free survival of GC patients, thereby reducing ASMR and ASDR (25–27). Finally, compared to the high incidence regions of East Asia, the USA has a significant advantage in terms of public health knowledge and education, which is a critical factor in the decline of GC incidence. The American public’s awareness of Hp infection and its association with GC has led to proactive preventive measures, effectively lowering Hp infection rates and thereby reducing the incidence of GC. Data indicate that the Hp infection rate in the USA is approximately 36%, well below the global average of 50% (28, 29). The World Health Organization has classified Hp as a Group 1 carcinogen, establishing a clear causal relationship with non-cardia GC (30). Hp can persistently colonize the gastric mucosa, causing chronic inflammation that leads to DNA damage and promotes the development of GC (31). The significant progress made in the prevention, early diagnosis, and treatment of GC in the USA has been the primary driver behind the continued decline in GC-related disease burden. These advancements have not only reduced GC incidence and mortality rates but also provided valuable insights and lessons for global GC prevention and management.

Several studies have indicated that the incidence of GC in men is typically about twice that in women, a conclusion that aligns closely with the findings of our study (32). In our research, we observed that the male-to-female ratio for newly diagnosed and existing GC cases in the USA in 2021 was 1.5:1. Firstly, the daily smoking rate among men is approximately five times higher than that among women (25% vs. 5.4%), indicating that smoking is significantly more prevalent among men (33, 34). Smoking increases the risk of GC in men by 60%, particularly for cardia cancer. Tobacco carcinogens can directly damage the gastric mucosa or promote Hp infection, leading to chronic gastritis and thereby increasing the risk of GC (35). Secondly, female reproductive hormones such as estrogen and progesterone are thought to play a protective role in preventing the development of GC (36). Research suggests that estrogen may reduce the risk of GC through several mechanisms, including decreasing chronic inflammation caused by Hp infection, suppressing the expression of cancer-related genes, and enhancing the protective functions of the gastric mucosa. This hormonal protective effect partly explains the lower incidence of GC in women. Finally, male patients often have more comorbid factors, with alcohol consumption and high body mass index (BMI) being known significant risk factors for GC. Studies have shown that the risk of GC is 7% and 30% higher in individuals who drink alcohol and those with high BMI, respectively, compared to those without these risk factors (8, 37, 38). Therefore, developing more effective public health policies to control these risk factors is crucial for reducing the incidence of GC. Governments and public health organizations should increase awareness of the dangers of smoking and alcohol consumption and promote healthier lifestyle choices.

Additionally, we observed that individuals aged 60 and above are at a higher risk of developing GC. This increased risk is likely attributable to the cumulative effects of adverse lifestyle habits and carcinogenic environmental factors over many years, with the pathology often being well-differentiated, predominantly intestinal-type GC (39). In our study, we found that the disease burden associated with GC significantly increases with age, with the majority of this burden concentrated in individuals aged 60 and above. This finding underscores the profound impact of demographic aging on the overall trend of GC burden. Our analysis aligns with the conclusion that the rising burden of GC is closely linked to an aging population. Specifically, our research indicates that the burden of GC peaks in the age groups of 75-79 years for females and 70-74 years for males. Similarly, existing studies have noted that GC is more prevalent among middle-aged and older adults (40). With age, especially in people over 60 years old, the function of the immune system decreases significantly due to the interaction of many complex factors, which greatly increases the risk of GC. Relevant studies have shown that the risk of GC in people over 60 years old is more than two times that of adolescents. Firstly, immune senescence leads to a comprehensive degradation of innate and acquired immune functions, which significantly reduces the body’s ability to clear cancerous cells and defend against Hp infection (41). Secondly, the chronic low-grade inflammatory state, a common age-related phenomenon, provides an ideal microenvironment for tumorigenesis and progression through prolonged systemic inflammation (42). At the same time, older adults often face the dual challenges of malnutrition and chronic co-morbidities, and these health problems may also further weaken immune function through multiple pathways (43). These results collectively suggest that the USA should implement more targeted policies for the elderly population to effectively address the GC burden, particularly in the context of an increasingly aging society.

In this study, we examined the spatial correlations in GC incidence across various regions in the USA and investigated the spatial distribution characteristics of GC incidence rates. Our findings indicate that GC incidence is not uniformly distributed across the country; instead, it shows marked spatial clustering, with higher incidence rates in certain high-income regions. Firstly, the trend of population aging is more pronounced in economically developed regions, which tend to have a higher percentage of elderly population. Studies have shown that the incidence of GC is significantly higher among people aged 60 years and older, making aging one of the key drivers of the high incidence of GC in these regions. This demographic change may be one of the key factors behind the interregional differences in GC incidence, which is supported by the decomposition analysis in this study (44). Secondly, economically developed regions such as California and New York have attracted a large number of immigrants to settle in the region due to their high level of economic development and superior quality of life. California, in particular, is one of the fastest growing regions in the United States in terms of population. Relevant studies have shown that the incidence of GC in Hispanic patients is significantly higher in areas with large Hispanic populations (e.g., California and Texas), and thus these immigrants have contributed to some extent to the increase in the incidence of GC in economically developed areas as well. Population migration and diversity may also introduce different genetic susceptibility and lifestyle factors, further influencing the regional distribution characteristics of GC (45). Finally, the widespread use of endoscopic screening may lead to detection bias, thus increasing the incidence of GC in these regions. Developed regions have more advanced healthcare infrastructures and higher healthcare accessibility, which makes early detection and diagnosis of GC more common, which may explain, in part, the higher incidence rates reported in these regions (46). This underscores the importance of continuing efforts to ensure equitable distribution of healthcare resources.

In the coming years, the heavy burden of GC is expected to rise, necessitating cost-effective strategies. Firstly, raising national awareness by disseminating information on cancer prevention and treatment is crucial. Secondly, global warming has been linked to an increase in gastric cancer incidence, potentially due to environmental changes that affect food preservation and increase the spread of Hp - a known risk factor for gastric cancer. Incorporating climate change into public health strategies could help mitigate this rising risk (47). Thirdly, early screening and intervention remain key strategies to significantly enhance survival rates and quality of life. Additionally, leveraging machine learning models holds promise for improving early diagnosis of GC by identifying high-risk individuals and enabling more precise screening (48). Lastly, increasing investment in cancer research to enhance treatment options is both essential and urgent, as these advancements will contribute to reducing the long-term burden of gastric cancer.

However, this study has several limitations. Firstly, it relies on secondary data analysis from the GBD study, which comes with inherent challenges regarding data quality and reliability. Secondly, while many risk factors are linked to GC, we focused on only a select few for further exploration. Lastly, the predictive models used offer a broad perspective on future trends but are not capable of providing exact forecasts. Addressing these limitations will require translating research findings into actionable steps, such as developing public policies and guiding future research efforts.

## Conclusion

GC remains a significant public health issue in the USA. Although the ASIR, ASPR, ASMR, and ASDR for GC are declining, the absolute number of cases and disease burden remain substantial, particularly among men and individuals over 60 years old. To address this persistent burden, targeted public health strategies are essential. These include improving awareness of GC risk factors such as Hp infection and unhealthy dietary habits, expanding early screening programs for high-risk populations, and promoting healthy lifestyles to mitigate modifiable risks. Additionally, optimizing healthcare resource allocation to ensure equitable access to prevention and treatment services is crucial, particularly for underserved groups. By implementing these measures, the burden of GC in the USA can be significantly reduced, leading to improved outcomes and public health.

## Data Availability

The original contributions presented in the study are included in the article/**Supplementary Material**. Further inquiries can be directed to the corresponding author.
